# A combination of multiple autoantibodies is associated with the risk of Alzheimer’s disease and cognitive impairment

**DOI:** 10.1038/s41598-021-04556-2

**Published:** 2022-01-25

**Authors:** Sung-Mi Shim, Young Ho Koh, Jong-Hoon Kim, Jae-Pil Jeon

**Affiliations:** 1 Division of Brain Disease Research, Department of Chronic Disease Convergence Research, Korea National Institute of Health, Osong-eup, Chungcheongbuk-do 28159 Republic of Korea; 2 Department of Biotechnology, College of Life Sciences and Biotechnology, Korea University, Seoul, 02841 Republic of Korea; 3 Present Address: Division of Biobank, Department of Precision Medicine, Korea National Institute of Health, Osong-eup, Chungcheongbuk-do 28159 Republic of Korea

**Keywords:** Alzheimer's disease, Biomarkers

## Abstract

Autoantibodies are self-antigen reactive antibodies that play diverse roles in the normal immune system, tissue homeostasis, and autoimmune and neurodegenerative diseases. Anti-neuronal autoantibodies have been detected in neurodegenerative disease serum, with unclear significance. To identify diagnostic biomarkers of Alzheimer’s disease (AD), we analyzed serum autoantibody profiles of the HuProt proteome microarray using the discovery set of cognitively normal control (NC, n = 5) and AD (n = 5) subjects. Approximately 1.5-fold higher numbers of autoantibodies were detected in the AD group (98.0 ± 39.9/person) than the NC group (66.0 ± 39.6/person). Of the autoantigen candidates detected in the HuProt microarray, five autoantigens were finally selected for the ELISA-based validation experiment using the validation set including age- and gender-matched normal (NC, n = 44), mild cognitive impairment (MCI, n = 44) and AD (n = 44) subjects. The serum levels of four autoantibodies including anti-ATCAY, HIST1H3F, NME7 and PAIP2 IgG were significantly different among NC, MCI and/or AD groups. Specifically, the anti-ATCAY autoantibody level was significantly higher in the AD (*p* = 0.003) and MCI (*p* = 0.015) groups compared to the NC group. The anti-ATCAY autoantibody level was also significantly correlated with neuropsychological scores of MMSE (r_s_ = − 0.229, *p* = 0.012), K-MoCA (r_s_ = − 0.270, *p* = 0.003), and CDR scores (r_s_ = 0.218, *p* = 0.016). In addition, a single or combined occurrence frequency of anti-ATCAY and anti-PAIP2 autoantibodies was significantly associated with the risk of MCI and AD. This study indicates that anti-ATCAY and anti-PAIP2 autoantibodies could be a potential diagnostic biomarker of AD.

## Introduction

Autoantibodies are reactive to self-antigens. They are known to be ubiquitous in all humans and implement adaptive debris-clearance for homeostasis and immune tolerance^1^. However, a variety of autoantibodies are likely to be involved in the pathogenesis of autoimmune diseases like rheumatoid arthritis^2^, chronic diseases like diabetes^3^, cancers^4^, or neurodegenerative disease like Parkinson’s disease^5^, Alzheimer’s disease (AD)^6^, or multiple sclerosis^7^. Autoantibodies in these diseases have been investigated as blood based-biomarkers for the prediction or diagnosis of disease as well as functional roles in disease pathology.

AD is a neurodegenerative disorder that is accompanied by deteriorated memory and cognitive function. The predominant factor in AD pathogenesis is amyloid-beta accumulation in the brain. However, it has been reported that inflammation and autoimmunity might contribute to AD development^8,9^. Autoantibodies against various antigens, including amyloid-beta (Aβ), neurotransmitters, microglia, lipids, and vascular-related molecules, have been considered autoimmune factors related to AD, suggesting their potential as diagnostic biomarkers or therapeutic agents for AD^6,7^. The most well-known autoantibody in AD is Aβ autoantibody. Anti-Aβ autoantibodies were found to be reduced in the serum of patients with AD^10,11^ and were considered protective against Aβ toxicity by catalyzing Aβ peptide and inhibiting the aggregation of Aβ^12–15^. In addition, autoantibodies against glutamate, neurotransmitter^16^, oxidized low-density lipoproteins^17^, and angiotensin 2 type 1 receptor were increased in the serum or cerebrospinal fluid (CSF) of patients with AD^18^. Autoantibodies derived from serum react with the brain and weaken the blood–brain barrier (BBB)^19,20^. Furthermore, the brain-reactive autoantibodies pass through the BBB, combine with neuronal surface molecules, and enhance Aβ penetration and deposition into neuronal cells, possibly inducing cell death^20^. Although autoantibodies against molecules related to AD pathology have been discovered, they have not yet been used as clinical diagnostic biomarkers of AD. Autoantibody profiling analysis using protein microarrays has been proposed as a tool to screen new autoantibodies for AD diagnosis or progression^21–23^. Autoantibody profiling in patients with AD identified various antigenic proteins such as SOS1, TNFRSF21, ATM, S100A1, PTCD2, or FRMD8, which were up-regulated in the blood of patients with AD and showed that the use of autoantibody panels or combinations elevated the possibility of AD diagnosis^22,23^. Considerable evidence has indicated an association between autoantibodies and AD, but their significance remains unknown.

We aimed to identify AD-related autoantibodies and evaluate their potential as biomarkers to predict or diagnose AD or mild cognitive impairment (MCI). We profiled autoantibodies in the serum of patients with AD and cognitively normal healthy controls using HuProt proteome microarray. Finally, five autoantibodies were investigated to determine their specificity in AD and MCI using enzyme-linked immunosorbent assay (ELISA).

## Results

### Global autoantibody profile using proteome microarray

**Figure 1:**
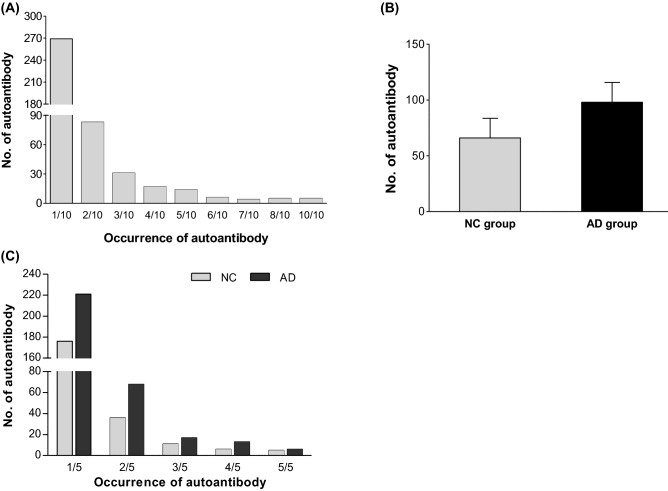
Autoantibody occurrence in AD patients (AD) and cognitively normal control subjects (NC). (**A**) Autoantibody occurrence in all tested subjects. Autoantibody occurrence indicates the frequency as to how many times a certain autoantibody is detected in 10 subjects of the discovery set. The most autoantibodies were detected in only one subject with an occurrence of 1/10. (**B** and **C**) Autoantibody occurrence in the individual AD and NC groups. Bars show mean ± standard deviation (SD).

The HuProt proteome microarray was used to screen the autoantibody profiles of selected serum samples from older adult Koreans (n = 10), including five AD patients and five cognitively normal controls (NC). The proteome microarray experiment showed positive signals corresponding to a total of 434 autoantibodies (n = 82.0 ± 41.1 autoantibodies per person), excluding immunoglobulin signals. Approximately two-thirds (n = 269 autoantibodies) of autoantibodies represented participant specific signals with an occurrence in one of the ten participants, while only five autoantibodies occurred in all ten participants (Fig. 1A, Supplementary Table 1).

### AD-related autoantibody profile

We compared autoantibody profiles from patients with AD (n = 5) and cognitively NC subjects (n = 5) to identify AD-associated autoantibodies. The AD group exhibited more autoantibodies (98.0 ± 39.9 autoantibodies per person) than those of the NC group (66.0 ± 39.6 autoantibodies per person) (Fig. 1B). In addition, all occurrences of autoantibodies in AD group were higher than those in NC group (Fig. 1C). Of the autoantibodies (n = 434) detected in the HuProt microarray experiments, 47 autoantigen signals were classified to AD-abundant signals that exhibited a higher occurrence with more than two of occurrence difference (delta > 2) in the AD group compared to the NC group (Supplementary Table 2). Especially, four autoantigen signals (e.g., PAIP2, HIST1H3F, C4orf40 and SURF5) showed the most AD-abundance with an occurrence difference of three (delta > 3), as shown in Supplementary Table 2.

The functional analysis using the DAVID (Database for Annotation, Visualization, and Integrated Discovery) revealed that 47 AD-abundant autoantibodies were annotated to antigen proteins enriched in non-membrane-bound organelles and the cytoskeleton in the GO cellular component as well as the ErbB signaling pathway in the KEGG pathway (Supplementary Tables 3 and 4).

### Validation of autoantibody candidates

To verify candidate autoantigen signals detected in the HuProt proteome microarray experiment, in total 12 autoantigens were selected according to three criteria of (1) AD-abundant, (2) ubiquitous, or (3) arbitrarily selected autoantigens. We also assessed the correlation between the microarray intensity and ELISA measurements of these 12 autoantigen candidates in the discovery sample set (n = 10). Five autoantigen candidates (ATCAY, HIST1H3F, NME7, NOL3, and PAIP2) showed a significant correlation between the two features of the HuProt and ELISA experiments (Supplementary Table 5).

**Figure 2:**
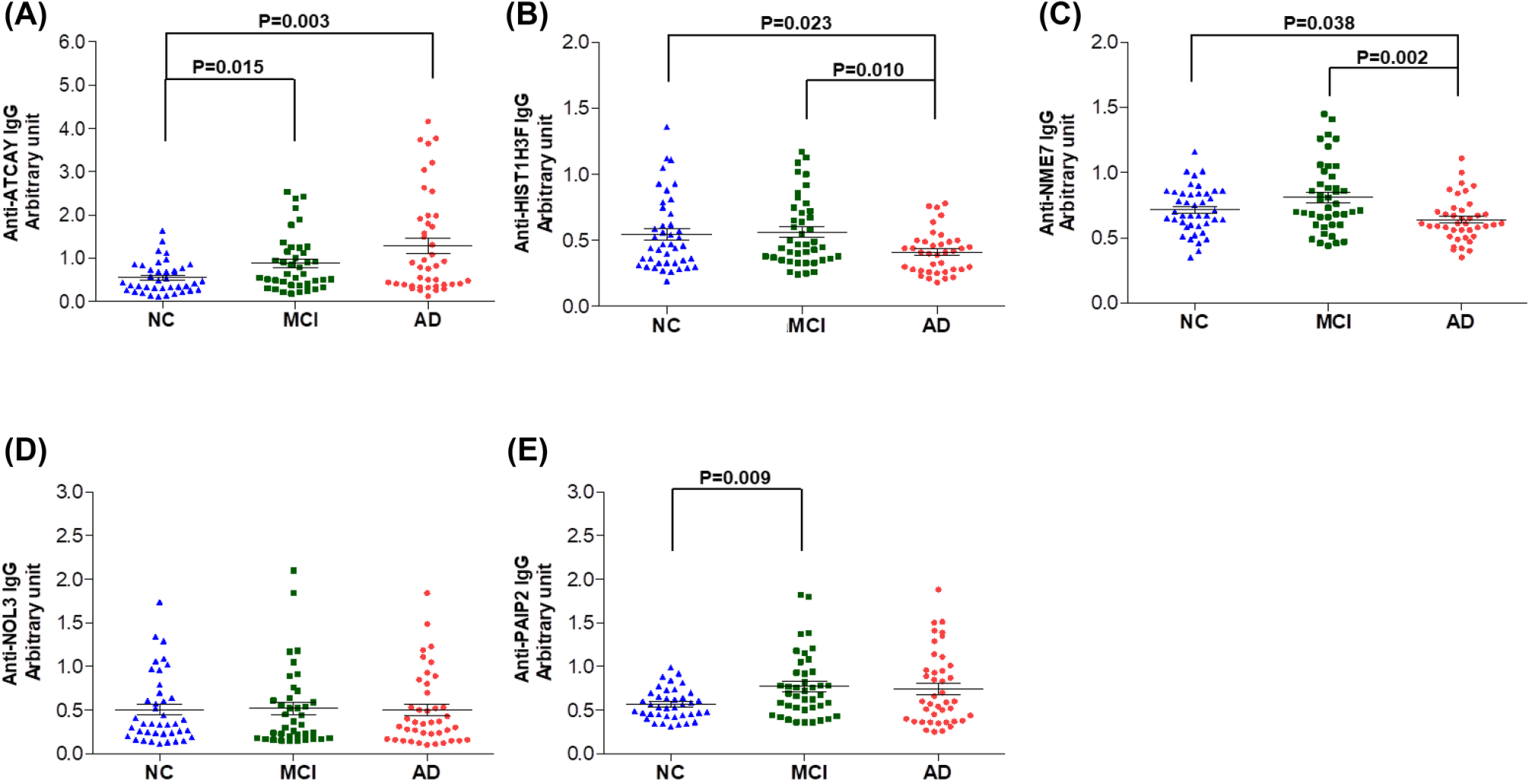
Levels of serum autoantibodies in the AD, MCI and cognitively normal control groups. Serum levels of anti-ATCAY IgG (**A**), anti-HIST1H3F IgG (**B**), anti-NME7 IgG (**C**), anti-NOL3 IgG (**D**), and anti-PAIP2 IgG (**E**) were indicated as an arbitrary unit which was defined as optical densities normalized by signals from the human whole IgG. Statistical significance was calculated by Mann–Whitney test after removing outliers that were more than 1.5 × interquartile range (IQR). Bars show mean ± standard error of the mean (SEM).

Next, serum levels of these five antibodies were measured using indirect ELISA in the extended validation sample set (n = 132; n = 44 for NC, n = 44 for MCI, and n = 44 for AD) to validate whether the autoantibodies could be used as biomarkers for MCI and AD. Statistical analysis of autoantibody measurements without outliers showed that the levels of anti-ATCAY IgG, anti-HIST1H3F IgG, anti-NME7 IgG, and anti-PAIP2 IgG autoantibodies were significantly different among the three groups (*p* = 0.005, *p* = 0.019, *p* = 0.005, and *p* = 0.050, respectively; Table 1). In particular, anti-ATCAY levels were significantly higher in the AD (*p* = 0.003) and MCI group (*p* = 0.015) than the NC group (Fig. 2A). Anti-HIST1H3F and anti-NME7 autoantibody levels were significantly lower in the AD group than the MCI (*p* = 0.010 and *p* = 0.002, respectively) and NC group (*p* = 0.023 and *p* = 0.038, respectively) (Fig. 2B,C). In addition, anti-PAIP2 IgG level was higher (*p* = 0.009) in the MCI group than the NC group (Fig. 2E) whereas anti-NOL3 IgG level were not different among three groups (Fig. 2D).

**Table 1:** Levels of autoantibodies in the AD, MCI and NC group.

Autoantibodies	NC	MCI	AD	*p value*^a^
anti-ATCAY IgG	0.55 ± 0.36	0.88 ± 0.66	1.28 ± 1.16	0.005
anti-HIST1H3F IgG	0.55 ± 0.28	0.56 ± 0.26	0.41 ± 0.16	0.019
anti-NME7 IgG	0.72 ± 0.18	0.81 ± 0.27	0.64 ± 0.17	0.005
anti-NOL3 IgG	0.51 ± 0.40	0.52 ± 0.46	0.50 ± 0.42	0.952
anti-PAIP2 IgG	0.57 ± 0.18	0.77 ± 0.37	0.75 ± 0.42	0.050

Data are shown as mean ± standard deviation (SD).

^a^Significant differences were evaluated by Kruskal–Wallis test after removing outliers that were more than 1.5 × interquartile range (IQR) of autoantibody measurements.

### Association between autoantibody profile and AD risk factors

ApoE4 is the major genetic risk factor of AD. In order to assess if ApoE4 is associated with autoantibody profiles, we examined a difference in the number of autoantibodies detected in the proteome chip between ApoE4 and non-ApoE4 carriers. The subjects in this study were divided into ApoE4 and non-ApoE4 carriers according to the presence of ApoE alleles. For example, ApoE4 carriers were defined as subjects with at least one allele of ApoE4 such as E2/E4, E3/E4, or E4/E4, and non-ApoE4 carriers with the absence of E4 allele. A significantly higher number of autoantibodies were detected in ApoE4 carriers (100.3 ± 34.8, *p* = 0.003) than non-ApoE4 carriers (39.3 ± 8.0). However, in terms of serum levels of autoantibodies, the only anti-PAIP2 autoantibody exhibited marginally significant difference (*p* = 0.049) in autoantibody levels between ApoE4 and non-ApoE4 carriers (Supplementary Tables 6 and 7). This result suggests that ApoE4 is implicated in autoantibody generation. In addition, it is known that the AD prevalence is higher in females than males. Thus, we tested a gender-difference of autoantibody levels among NC, MCI and AD groups. The anti-HIST1H3F (*p* = 0.037) and anti-NOL3 (*p* = 0.041) autoantibody levels were significantly higher in females than males only in the AD group of the validation sample set (Supplementary Table 8). This result suggests that a gender difference of some AD-related autoantibodies may provide a clue to understand a higher prevalence of AD in females than males. Amyloid and tau are well-known pathological factors of Alzheimer’s disease. Instead of these antigens, autoantibodies against total Tau (tTau) and phosphorylated Tau (pTau) were tested to expand autoantibody profiles of AD using the validation samples. There was no significant difference of anti-tTau and -pTau IgG autoantibody levels among NC, MCI and AD groups (Supplementary Table 9). In addition, these anti-Tau IgG autoantibodies were not also correlated with any of five autoantibodies (data not shown).

### Association between IgG autoantibodies and total IgG levels

We measured total IgG levels using direct ELISA to determine whether total IgG levels affect autoantibody levels. There was no difference in total IgG levels among AD, MCI, and NC participants (Supplementary Fig. 1). However, the concentration of total IgG was significantly positively correlated with each level of the five autoantibodies, with the highest correlation (r_s_ = 0.473, *p* < 0.001) with anti-NME7 IgG levels (Supplementary Table 10). Based on these findings, we compared the autoantibody levels among the three groups after normalizing the autoantibody level by total IgG levels. When normalized by the total IgG level, the anti-ATCAY IgG autoantibody was detected in a significantly higher level in the MCI (*p* = 0.021) and AD (*p* = 0.002) groups than NC groups (Supplementary Fig. 2A). Levels of total IgG-normalized anti-PAIP2 IgG autoantibody showed the significant difference between the MCI and NC groups (*p* = 0.027, Supplementary Fig. 2E), but not for the other autoantibodies (Supplementary Fig. 2B-D).

### Autoantibody occurrence in AD, MCI and NC

We investigated the occurrence of five autoantibodies in the AD, MCI, and NC groups to assess the association between autoantibody occurrence frequency and dementia. The detection cut-off of autoantibodies was set as the mean plus 2 standard deviations (SD) of autoantibody signal intensities in the NC group. The individual anti-ATCAY IgG and anti-PAIP2 IgG exhibited a significantly higher occurrence frequency in MCI and AD groups than the NC group (Table 2). Study participants who were positive for either anti-ATCAY IgG or anti-PAIP2 IgG were found to be at an increased risk of MCI (OR = 6.17, 95% CI = 1.25–30.32 and OR = 11.40, 95% CI = 1.37–95.04, respectively) and AD (OR = 10.67, 95% CI = 2.25–50.71 and OR = 17.03, 95% CI = 2.11–137.80, respectively). In contrast, the other autoantibodies (anti-HIST1H3F, anti-NME7, anti-NOL3) did not exhibit significant association of the autoantibody occurrence with MCI and/or AD (Table 2).

**Table 2:** Occurrence and risk assessment of a single autoantibody or the combined autoantibodies for the diagnostics of MCI and AD.

Type of autoantibody	NC	MCI	AD	NC_MCI		NC_AD	
	No. (%)	No. (%)	No. (%)	*p value*^a^	OR (95% CI)	*p value*^a^	OR (95% CI)
anti-ATCAY IgG	2 (5.1)	10 (25.0)	15 (36.6)	0.025	6.17 (1.25–30.32)	0.001	10.67 (2.25–50.71)
anti-HIST1H3F IgG	2 (4.9)	3 (7.1)	0 (0.0)				
anti-NME7 IgG	1 (2.3)	4 (9.5)	0 (0.0)				
anti-NOL3 IgG	3 (7.5)	6 (14.6)	8 (19.0)				
anti-PAIP2 IgG	1 (2.6)	9 (23.1)	13 (31.0)	0.014	11.40 (1.37–95.04)	0.001	17.03 (2.11–137.80)
anti-ATCAY or PAIP2 IgG	3 (6.8)	17 (38.6)	25 (56.8)	0.001	8.61 (2.30–32.21)	< 0.001	17.98 (4.83–67.00)

The cutoff value for positive autoantibody levels (normalized by total IgG) was set as mean plus 2 standard deviations (SD) of NC.

^a^Significance differences for occurrence of autoantibodies between groups were evaluated by Chi-square test or Fisher exact test, as appropriate.

Next, we tested whether the occurrence frequency of combinations of multiple autoantibodies increased the diagnostic power of AD and MCI. The combination of occurrence frequencies of anti-ATCAY and anti-PAIP2 autoantibodies increased the statistical power of the diagnosis of MCI (*p* = 0.001, OR = 8.61, 95% CI = 2.30–32.21) and AD (*p* < 0.001, OR = 17.98, 95% CI = 4.83–67.00) (Table 2). This result suggests that a single or combined occurrence frequency of anti-ATCAY and anti-PAIP2 autoantibodies can be a potential diagnostic biomarker of MCI and AD.

### Association of anti-ATCAY IgG autoantibody levels with cognitive function

**Figure 3:**
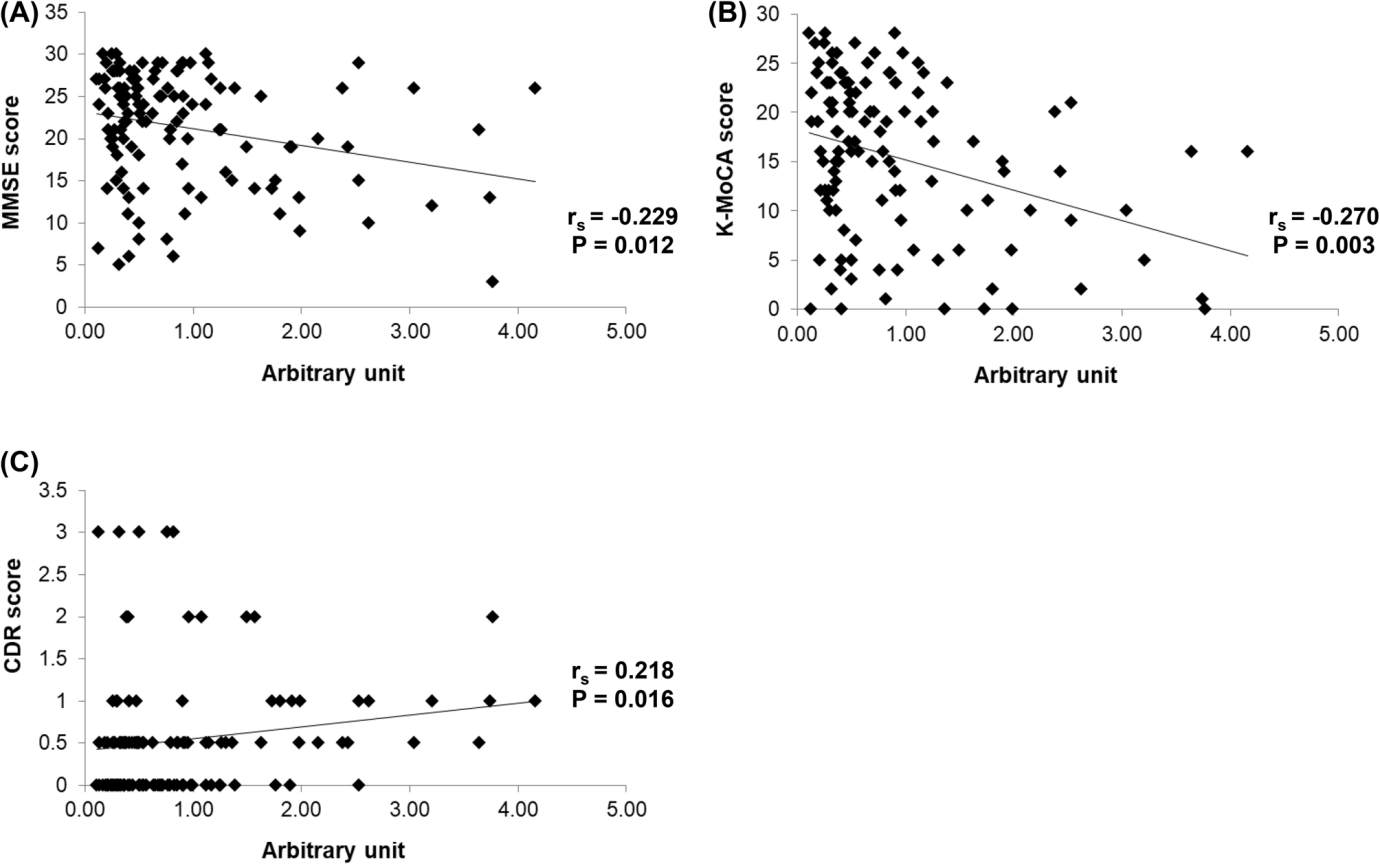
Significant association between anti-ATCAY IgG level and cognitive function. The anti-ATCAY IgG level was negatively correlated with MMSE scores (r_s_ = -0.229, *p* = 0.012) (**A**), negatively correlated with K-MoCA scores (r_s_ = − 0.270, *p* = 0.003) (**B**), and positively correlated with CDR scores (r_s_ = 0.218, *p* = 0.016) (**C**). The correlation was evaluated by Spearman correlation test after excluding the outliers of more than 1.5 × interquartile range (IQR).

To examine whether autoantibody levels are associated with cognitive function, we evaluated the correlation between autoantibody levels and neuropsychological scores, including the Mini-Mental State Examination (MMSE), the Korean version of the Montreal Cognitive Assessment (K-MoCA), and Clinical Dementia Rating Scale (CDR). When we re-tested associations of anti-ATCAY IgG autoantibody with cognitive function after removing the outliers, the anti-ATCAY IgG level was significantly negatively correlated with the MMSE (r_s_ = − 0.229, *p* = 0.012, Fig. 3A) and K-MoCA (r_s_ = − 0.270, *p* = 0.003, Fig. 3B) scores, but positively correlated with CDR (r_s_ = 0.218, *p* = 0.016, Fig. 3C), indicating a higher serum level of anti-ATCAY IgG in subjects with cognitive decline.

### Level of anti-ATCAY IgM autoantibody in AD

We also tested the levels of anti-ATCAY IgM autoantibody in the validation set to determine whether anti-ATCAY IgM can differentiate AD patients from NC individuals. We did not observe a difference in the levels of anti-ATCAY IgM autoantibody among the three groups (*p* = 0.346, Supplementary Fig. 3A). However, the anti-ATCAY IgM levels were positively correlated with the levels of anti-ATCAY IgG (r_s_ = 0.248, *p* = 0.004, Supplementary Fig. 3C), and the anti-ATCAY IgG/IgM ratio was higher in patients with AD than that in the NC group (Supplementary Fig. 3B).

## Discussion

This study found that autoantibodies were more abundant in older people with cognitive impairment than that in NC individuals. Through the proteome microarray and subsequent ELISA-based validation experiments using general population-based geriatric cohort samples, we discovered potential diagnostic autoantibodies (anti-ATCAY, -HIST1H3F, -NME7 and -PAIP2 IgG) of AD. In addition, the combined occurrence frequency of anti-ATCAY IgG and anti-PAIP2 IgG autoantibodies potentiated the diagnostic power of discriminating patients with AD from NC group. In contrast to the IgG autoantibody, the frequency of anti-ATCAY IgM autoantibody did not differ among patients with AD or MCI and NC groups, suggesting the the anti-ATCAY autoantibody detected in patients may be resulted from a chronic exposure of ATCAY autoantigen to the immune system rather than a short-term or acute exposure^24,25^. Taken together, these results suggest that combinations of serum autoantibodies can be used as potential biomarkers for AD diagnosis.

Here, we report, for the first time, that the anti-ATCAY autoantibody is a potential biomarker of MCI and AD. ATCAY was identified in Cayman cerebellar ataxia patients with mutations in the gene, a causative factor of the disease^26^. The gene encodes caytaxin (also called BINP-H) expressed in the brain, especially in the cerebellum and hippocampus^27,28^. The function of caytaxin in the brain and neuronal cells is not well-known. A few studies have reported that caytaxin includes the CRAL-TRIO domain, which interacts with lipophilic molecules^29^ and needs to maintain a balance of glutamate in the central nervous system by coupling with glutaminase and moving to the end of neurites^29^. In addition, caytaxin seems to be involved in transporting mitochondria to neurite terminals. The distribution of the complexes of caytaxin and the associated molecules to the neurite end is achieved by binding caytaxin to a motor protein, kinesin^30^. Caytaxin interacts with peptidyl-prolyl cis–trans isomerase NIMA-interacting 1 (Pin1), instead of glutaminase, on the differentiation of neurons induced by neuron growth factors, suggesting that post phosphorylation of caytaxin may be implicated in neuronal differentiation^31^. Altogether, caytaxin is likely to function in neuronal differentiation and brain development. However, the function of ATCAY in neurodegeneration has not yet been reported. Further studies are needed to investigate the role of caytaxin in neurodegeneration and AD, and how to generate ATCAY autoantibody.

HIST1H3F is a core protein of histone clusters that are responsible for composition and structure of nucleosome. HIST1H3F gene expression was reported to be up-regulated in hippocampus of mice with sleep deprivation^32^. However, the association between HIST1H3F and neurodegenerative diseases has not yet been known. NME7 is a member of the NME/NM23 family, which encodes nucleoside diphosphate kinases to generate nucleoside triphosphate. A recent report showed that NME7 was downregulated in the AD brain, resulting in the deregulation of purine metabolism^33^. NOL3 is an anti-apoptotic protein, known as an apoptosis repressor with a caspase recruitment domain (ARC) to inhibit caspase-3 and 8, and is increased in the frontal cortex of patients with AD compared with NC^34^. These findings for NME7 and NOL3 in AD are involved in AD pathology, but their autoantibodies have not yet been reported in patients with AD or MCI. Thus, more evidence is required to show the relationship between these autoantibodies and AD.

PAIP2 is a translational repressor that displaces poly (A)-binding protein (PABP) from mRNA. In the brain, PAIP2 was reported to inhibit the translation of CaMKIIa mRNA, which is involved in memory generation, resulting in control of synaptic plasticity and memory^35^. The present study showed that the level of PAIP2 IgG autoantibody was significantly higher in the MCI group but not in the AD group than the NC group. In contrast, an earlier study proposed that the PAIP2 autoantibody was prevalent in NC compared to patients with AD^22^. This inconsistency may be due to a different ethnicity and small sample size, and then could be resolved from the future replication studies using larger samples from clinically well-characterized AD patients.

This study showed that IgM autoantibodies against ATCAY were present in all serum samples, but their levels were not significantly different among AD, MCI, and cognitively NC groups, in contrast to anti-ATCAY IgG. A functional role of IgM autoantibody in AD has been reported as a catalytic factor of Aβ^13^. However, Marcello et al.^36^ did not observe a significant difference in the levels of complex IgM and Aβ among AD, MCI, and NC groups. In the acquired immune system, IgM is generated in the primary response against foreign antigens and disappears within several weeks. On the other hand, IgG replaces IgM to participate in the secondary response and maintains long-term immunity^24,25^. Pathogenic condition in AD may activate class switching from IgM to IgG production^23^. A possible explanation for our results is that IgM autoantibodies may be involved in the early stage of AD, and their roles may be turned over to IgG during disease progression. Although IgG autoantibodies in AD are primarily abundant among Ig isotypes, evidences for association between AD and other Ig isotypes such as IgM, IgA, or IgG subclasses (IgG1, IgG2, IgG3, IgG4) are growing^37–39^. In addition, the identification of immune cells producing the AD-related autoantibodies would support a strong link between AD and autoantibodies^40^. Therefore, it needs to be further investigated how autoantibody profiles in different Ig isotypes are changed and how immune cells, including B cell and T cell, are involved in the progression of AD.

One of the risk factors of AD pathogenesis is neuroinflammation or neurovascular dysfunction. In our present study, autoantibodies against any inflammatory proteins were not detected. One study reported that there were brain-reactive autoantibodies in serum, and the autoantibodies promoted the intraneuronal accumulation of Aβ42 in AD brain^41^. The brain-reactive autoantibodies in serum would flow into the brain by breakdown of blood–brain barrier (BBB) whose permeability could be elevated in an age-dependent manner^42,43^. In addition, autoantibodies against brain-specific molecules can be generated when such molecules are exposed to blood immune systems^44^. An influx of blood-derived molecules including autoantibodies, peripheral immune cells, or pathogens could result in an imbalance of the immune system and neuroinflammation in the brain, preventing Aβ clearance and accelerating Aβ deposition^45^. The neuroinflammation is suggested to be directly involved in the pathogenesis of AD^46^. The generation and role of autoantibodies in AD seems to be linked to neurovascular dysfunction. Therefore, it is speculated that ATCAY-like brain specific proteins may be newly exposed to the blood immune system due to BBB breakdown, generating autoantibodies in the AD pathogenesis^44^. There are a growing number of literatures indicating that natural autoantibodies play a protective or pathogenic role in the pathogenesis of neurodegenerative diseases including AD^47,48^. Our results suggest that autoantibody repertoires are changing in the AD pathogenesis, supporting that AD is a autoantibody-related autoimmune disorder. Changes in autoantibody repertoires may be possibly due to brain injury or neuroinflammation^49^, but further studies are still needed.

In our study, combinations of autoantibodies provided a higher sensitivity for predicting the risk of AD than a single autoantibody. Consistent with previous studies, autoantibody panels identified by the proteomic microarray approach showed high accuracy in discriminating AD or MCI from NC or other diseases^21,22^. Therefore, the combinations or panels of previously reported autoantibodies will increase the statistical power of biomarkers to predict or diagnose AD.

This study has several limitations. (1) Small sample size: The use of a very small number of samples in the proteome microarray for the autoantibody screening stage resulted in the detection of a lower number of ubiquitous or AD-associated autoantibodies compared to earlier studies^22,50^. Thus in the subsequent validation stage, we tested candidate autoantibodies in a larger number of age- and gender-matched samples to overcome this weakness. (2) Clinically not well-annotated samples: In the present study, study subjects were not specifically tested for Aβ pathology (amyloid-PET, Aβ40 and Aβ42 in CSF), Tau pathology (total or phosphorylated tau levels in CSF), and neurodegeneration/neuronal injury. According to some reports^51,52^, a substantial percentage (12~23%) of AD-diagnosed patients were definitely found to be mis-diagnosed or Alzheimer's disease mimics at the post-mortem neuropathological diagnosis. Thus, it is very important to use high-quality and well-annotated biological samples of true AD excluding Alzheimer's disease mimics, allowing for the development of precise predictive biomarkers and therapeutic drugs of Alzheimer's disease. Generally, the true AD patients can be definitely diagnosed at autopsy. Therefore, this study result should be replicated in other independent cohorts or larger populations with well-characterized subtypes of AD. (3) Lack of disease specificity: We did not determine whether the autoantibodies could specifically discriminate AD or MCI from other neurodegenerative diseases such as frontotemporal dementia (FTD), Parkinson’s disease (PD), or amyotrophic lateral sclerosis (ALS). (4) Exclusion of subjects with comorbidities in the discovery set: We selected the discovery set which excluded subjects with comorbid conditions such as diabetes and hypertension, while the validation set included age- and gender-matched groups of NC, MCI and AD participants who were selected regardless of comorbidities such as diabetes, hypertension, depression, and cardiovascular diseases. Chronic diseases including diabetes, hypertension, depression, and cardiovascular disease have been known to affect the development of AD^53^. Thus, AD-related autoantibodies need to be tested for sub-groups of AD patients with or without co-existing diseases.

In conclusion, autoantibodies are known to be involved in AD through various AD studies, including the present study. We discovered AD or MCI-associated autoantibodies against ATCAY and PAIP2 antigens, and observed significantly higher occurrence of anti-ATCAY and anti-PAIP2 IgG autoantibodies in MCI and AD. Moreover, combinations of multiple autoantibodies were shown to be more powerful for evaluating the risk of MCI and AD. Our results support that AD-associated autoantibodies can be used as potential biomarkers for the diagnosis of AD. In addition, these findings also support that AD is a autoantibody-related autoimmune disorder.

## Methods

### Study participants

Serum samples were collected from older adult Koreans who participated in the general population-based geriatric cohort study from 2009 to 2010^54,55^. The discovery set consisted of ten samples from age- and gender-matched five AD patients and five cognitively normal subjects who did not have both diabetes and hypertension in order to preclude a possibility of metabolic disease-related autoantibodies. In contrast, the validation set consisted of age (± 2)- and gender-matched groups of NC (n = 44), MCI (n = 44) and AD (n = 44) participants who were selected regardless of comorbidities such as diabetes, hypertension, depression, and cardiovascular diseases. The demographic characteristics of the study participants are summarized in Table 3. Cognitive function and memory decline were evaluated using the Korean version of the Consortium to Establish a Registry for AD (CERAD-K) neuropsychological test battery^56^. Dementia was diagnosed according to the guidelines of the Diagnostic and Statistical Manual of Mental Disorders, fourth edition (DSM-IV)^57^. The CDR was assessed on the basis of structured interviews with caregivers. The CDR scores were obtained in six functional domains such as memory, orientation, judgment and problem solving, community affairs, home and hobbies, and personal care^58,59^. MCI was diagnosed according to the Petersen/Winblad criteria^60,61^. In addition, K-MoCA was used for the detection of MCI^62^. The research protocols were approved by the Institutional Review Board (IRB) of Korea Centers for Disease Control and Prevention (KCDC) (IRB approval number: 2016-02-23-P-A, 2017-05-05-P-A), and experiments were performed in compliance with the relevant regulations and guidelines. Written informed consent was obtained from the study participants after explanation of the study design and protocols.

**Table 3:** Demographic data of study participants.

	Discovery set			Validation set			
	NC	AD	*p* value^a^	NC	MCI	AD	*p* value^b^
Individuals, n (F/M, gender)	5 (5/0)	5 (5/0)	−	44 (36/8)	44 (36/8)	44 (36/8)	−
Age, years	77.8 ± 2.2	79.2 ± 2.2	−	75.9 ± 5.8	75.8 ± 5.8	76.0 ± 5.9	−
Education, years	8.6 ± 5.0	2.2 ± 2.1	0.04	6.6 ± 4.8	4.3 ± 4.4	3.6 ± 4.5	0.01
MMSE	28.4 ± 2.5	15.4 ± 2.4	< 0.001	26.8 ± 2.5	22.9 ± 3.7	14.8 ± 5.9	< 0.001
CDR	0.0 ± 0.0	0.9 ± 0.2	0.001	0.1 ± 0.2	0.3 ± 0.3	1.2 ± 0.8	< 0.001
ApoE4 carrier (%)	40.0	100	−	11.6	31.0	40.9	−

Data are shown as mean ± standard deviation (SD).

^a^Significant differences were evaluated by Independent *t* test.

^b^Significant differences were evaluated by one-way ANOVA test.

### Human proteome microarray

We used the HuProt human proteome microarray version 2.0 (CDI Laboratories, Baltimore, MD, USA), including 19,275 individually purified human proteins on a 3D polymers-coated slide, to screen for autoantibodies. All proteins are printed in duplicate and tagged with N-terminal glutathione s-transferase (GST) and regulator of G-protein signaling (RGS)-His6. Serum samples from patients with AD (n = 5) and NC (n = 5) subjects were probed on an individual microarray according to the manufacturer’s protocol. Briefly, the microarray was blocked with 5% bovine serum albumin (BSA) in TBST (Tris-buffered saline pH 7.5, 0.1% Tween20) for 2 h at room temperature (RT). The microarray was probed with serum diluted 1:500 in 5% BSA in TBST and incubated with shaking for 1 h at RT. Then, the microarray was washed three times with TBST on a shaker for 10 min and incubated for 1 h after applying Cy5-labeled goat anti-human IgG (H + L) (Abcam, Cambridge, UK) diluted 1:1000 with 5% BSA in TBST. Next, the microarray was washed, dried at 800 rpm for 3 min, and scanned using a GenePix 4000 B fluorescence scanner (Molecular Devices, Sunnyvale, CA, USA).

### Proteome microarray data analysis

The scanned images were analyzed using GenePix Pro Software Version 6.0 (Molecular Devices, Sunnyvale, CA, USA, https://support.moleculardevices.com/s/article/ GenePix-Pro-6-Microarray-Acquisition-Analysis-Software-Download-Page). Signal intensities of spots were determined as the F median (the median of all the feature pixel intensities) minus the B median (the median of all the background pixel intensities). In the screening discovery stage, a certain probe signal was considered as a positive protein spot when the signal intensity was more than three sigma (3 standard deviations) of signal intensities for all probes on the microarray. For the discovery stage, we selected 12 proteins to validate the microarray results according to the following criteria. First, six AD-abundant autoantigen candidates (CHAC2, HIST1H3F, NOL3, PAIP2, RAB11FIP1, SURF5) were selected when the target spots showed positive signals in an occurrence frequency of more than three subjects of total five AD patients or simultaneously giving a difference of three in the occurrence frequency between the AD and NC groups (Supplementary Table 2). Second, five ubiquitous autoantigen candidates (ATCAY, CLC, GPBP1, SPANXN2, and TPM3) were selected when the target spots represented an occurrence frequency (> 7 over 10) of more than seven in all ten tested samples, regardless of AD or NC groups (Supplementary Table 1). The ATCAY autoantigen showed a positive signal in a frequency (8 over 10) of eight of 10 samples. Third, one particular autoantigen (NME7) was arbitrarily selected as a candidate biomarker that has been implicated in neurodegenerative diseases including AD. According to these three selection criteria as well as another key criterion of the commercial availability of recombinant antigen proteins, in total, 12 target autoantigen candidates were selected for the validation assay using ELISA.

### ELISA for measurement of autoantibodies and total IgG level in human

Serum levels of autoantibodies against autoantigens were measured using an indirect ELISA assay as follows. The wells of a 96-well plate (Thermo Scientific, Roskilde, Denmark) were coated with 100 ng of recombinant proteins (Supplementary Table 11 in coating buffer (0.05 M Carbonate/bicarbonate buffer, pH 9.6, SIGMA, Saint Louis, Mo, USA) and incubated at 4 °C overnight. After washing with PBST (0.05% Tween 20 in phosphate-buffered saline (PBS)), the wells were blocked with 5% skim milk in PBST and incubated at RT for 2 h. And then, 100 ul of diluted serum (1:50) in blocking buffer (1% BSA in PBS) was dispensed into the wells and incubated at RT for 2 h. Next, goat anti-human IgG H&L HRP antibody (Abcam, Cambridge, UK) or goat anti-human IgM mu HRP antibody (Abcam, Cambridge, UK) at 1:10,000 dilution with blocking buffer was dispensed into each well. Following further incubation at RT for 1 h, the wells were incubated with 100 µL of tetramethylbenzidine (TMB, Invitrogen, Camarillo, CA, USA) solution at RT for 15 min. The reaction was stopped by adding 100 µL of stop solution (Invitrogen, Vienna, Austria). The optical density (OD) was obtained at 450 nm using the microplate reader (SPECTRA MAX250, Molecular Devices, Silicon Valley, USA). All samples were measured in duplicate. Autoantibody signals were calculated by subtracting the background signal of the control wells (antigen-coated but no serum). The signals of autoantibodies derived from the human whole IgG (10 ug, GenScript, Piscataway, NJ, USA) as well as the pooled sera from NC subjects (n = 10) were measured as inter-assay controls in each assay to correct variation from plate to plate. The CVs of intra-assay and inter-assay for each autoantibody were below 6% and 10%, respectively. The levels of IgG autoantibodies against the tested autoantigens and anti-ATCAY IgM autoantibodies in subjects were expressed as arbitrary units normalized by the signal from the human whole IgG and pooled sera, respectively.

For the subsequent statistical analysis of autoantibody measurements obtained from the ELISA, we defined statistical outliers that were more than 1.5 × interquartile range (IQR) of levels of autoantibodies for each group. According to 1.5X IQR, we removed 8 ~ 14 outliers from the original ELISA measurements (n = 11 outliers for anti-ATCAY IgG, n = 8 for anti-HIST1H3F IgG, n = 8 for anti-NME7 IgG, n = 14 for anti-NOL3 IgG, n = 11 for anti-PAIP2 IgG). Specifically, there were 11 outliers (5 from NC, 4 from MCI, and 2 from AD) in anti-ATCAY IgG levels, 8 (2 form NC, 2 from MCI, and 4 from AD) in anti-HIST1H3F IgG levels, 8 (2 form NC, 2 from MCI, and 4 from AD) in anti-NME7 IgG levels, 14 (5 form NC, 6 from MCI, and 3 from AD) in anti-NOL3 IgG levels, and 11 (5 form NC, 3 from MCI, and 3 from AD) in anti-PAIP2 IgG levels. These outliers were not associated with MMSE, CDR, age, gender, and ApoE4 allele.

Serum concentrations of total IgG against particular autoantigens were measured using a direct ELISA as previously described^63^. In brief, 100 µL of diluted serum at 1:500,000 with the coating buffer were coated in the 96-well plate in duplicate for 2 h at RT. After washing with 300 µl of PBST three times, the wells were treated with 200 µL of the blocking buffer for 2 h at RT. Next, the goat anti-human IgG H&L HRP antibody at 1:10,000 dilution with the blocking buffer was applied at RT for 1 h, followed by addition of 100 µL of TMB solution. 100 µL of stop solution was dispensed into each well, and the OD at 450 nm was obtained using the microplate reader.

### Statistical analysis

All statistical analyses were performed using Statistical Package for Social Sciences Version 12.0 (SPSS Inc., Chicago, IL, USA, https://www.ibm.com/products/spss-statistics). The significance of differences in the autoantibody levels was assessed using the Mann–Whitney test or Kruskal–Wallis test between the AD, MCI, and NC groups. Correlations between autoantibody levels and clinical data were analyzed using the Spearman rank correlation test. The significance of autoantibody positivity was assessed using Pearson’s 2-tailed χ^2^ test or Fisher’s exact test.

## Supplementary Information

Supplementary Information.
