# Author Correction: A combination of multiple autoantibodies is associated with the risk of Alzheimer’s disease and cognitive impairment

**DOI:** 10.1038/s41598-022-06478-z

**Published:** 2022-02-07

**Authors:** Sung-Mi Shim, Young Ho Koh, Jong-Hoon Kim, Jae-Pil Jeon

**Affiliations:** 1 Division of Brain Disease Research, Department of Chronic Disease Convergence Research, Korea National Institute of Health, Osong-eup, Chungcheongbuk-do 28159 Republic of Korea; 2 Department of Biotechnology, College of Life Sciences and Biotechnology, Korea University, Seoul, 02841 Republic of Korea; 3 Present Address: Division of Biobank, Department of Precision Medicine, Korea National Institute of Health, Osong-eup, Chungcheongbuk-do 28159 Republic of Korea

Correction to: *Scientific Reports* 10.1038/s41598-021-04556-2, published online 25 January 2022

The original version of this Article omitted the Acknowledgements section. The Acknowledgements section now reads:

“This study was supported by intramural funds (2016-NG62003-00, 2017-NI62001-00) from Korea National Institute of Health (KNIH), the Korea Centers for Disease Control and Prevention (KCDC). Clinical and epidemiological data and biospecimens used in this study were obtained by an extramural research program (funded to Dr. Park MH) of KCDC, which was conducted in the Ansan Hospital of the Korea University Medical College.”

Furthermore, the Funding section in the original version of this Article was incomplete.

“Funding was provided by Korea National Institute of Health, the Korea Center for Disease Control and Prevention (Grant Nos. 2016-NG62003-00).”

now reads:

“Funding was provided by Korea National Institute of Health, the Korea Center for Disease Control and Prevention (Grant Nos. 2016-NG62003-00, 2017-NI62001-00).”

The original Article has been corrected.

