# Supplementary material for: A combination of multiple autoantibodies is associated with the risk of Alzheimer’s disease and cognitive impairment
Source: Sci Rep. 2022 Jan 25;12:1312. doi: 10.1038/s41598-021-04556-2 (PMC8789802; doi:10.1038/s41598-021-04556-2)
Supplement: Supplementary file 1 — Supplementary Information. [file 41598_2021_4556_MOESM1_ESM.docx]

**A combination of multiple autoantibodies is associated with the risk of Alzheimer’s disease and cognitive impairment**

Sung-Mi Shim^1§^, Young Ho Koh^1^, Jong-Hoon Kim^2^, Jae-Pil Jeon^1§*^

1 Division of Brain Disease Research, Department of Chronic Disease Convergence Research, Korea National Institute of Health, Osong, Korea

2 Department of Biotechnology, College of Life Sciences and Biotechnology, Korea University, Seoul, Republic of Korea

^*^ Correspondence should be addressed to Jae-Pil Jeon

187 Osongsaengmyung-2-ro, Osong-eup, Cheongju-si, Chungcheongbuk-do, Korea 363-951

Email: jaepiljeon@hanmail.net

Tel: 82-43-719-6550

Fax: 82-43-719-6539

§ Present affiliation: Division of Biobank, Department of Precision Medicine, KNIH

**Study subjects**

As originally described in Materials and Methods of the manuscript, the discovery set consisted of 10 study subjects including five pairs of age- and gender- matched AD patients and cognitively normal control subjects. Subjects with both diabetes and hypertension were excluded from the discovery set to preclude a possibility of metabolic disease-related autoantibodies. As shown in Table 3 of the manuscript, the AD patients of the validation set showed MMSE of 14.8 ± 5.9 and CDR of 1.2 ± 0.8, and 40.9% of APOE4 allele frequency. The discovery and validation sets were selected study subjects who participated in the forth-wave follow-up study of the Ansan Geriatric Study (AGE) cohort. Because the AGE was a general population-based geriatric cohort, there were only 48 AD patients of the forth-wave study participants (n=601). Therefore, almost all AD patients of the forth AGE participants were included in our validation set (n=44, AD patients), indicating that characteristics of the validation set are similar to those of the original cohort.

For more details of the original AGE cohort (Han et al, 2009, Shim et al, 2016): The AGE cohort included elderly Korean participants who were recruited for a general population-based geriatric cohort study. The AGE cohort was designed to establish a prospective population-based cohort to study prevalence, incidence, and related risk factors for geriatric diseases and to obtain comprehensive information on public health and functional status of elderly Koreans in urban community. At the pilot phase in 2003, a total of 2,767 elderly Koreans were enrolled to establish the baseline target population, aged 60-84 years. From these baseline target population, 1,391 subjects were randomly recruited for the first-wave study to perform comprehensive health examination including clinical and neuropsychological tests as well as biosample collection in 2004-2006. Subsequently in every two or three year interval, the second-, third-, and forth-wave follow-up examinations were conducted for 841, 600 and 601 study participants, respectively.

**HuProt microarray and ELISA experiments**

We used the HuProt human proteome microarray version 2.0 (CDI Laboratories, Baltimore, MD, USA), including 19,275 individually purified human proteins on a 3D polymers-coated slide, to screen for autoantibodies. All proteins are printed in duplicate and tagged with N-terminal glutathione s-transferase (GST) and regulator of G-protein signaling (RGS)-His6. We performed entire procedures from probing serum samples of patients with AD (n=5) and NC subjects (n=5) on a microarray to its scanning according to the manufacturer’s instruction. The method was described in the Materials and Methods section. Next, serum levels of autoantibodies against autoantigens were measured by indirect ELISA assay. For ELISA, optimum dilution ratios of serum samples were tested for each autoantibody in a dilution ratio of 1:20 ~ 1:100. We chose a dilution ratio of 1:50 giving 0.1~3.0 range of optical density (OD) at 450 nm because ODs at a dilution ratio of 1:20 in a few samples were close to 4.0 which is the cut-off value of OD. In all ELISA experiments, ELISA readings of samples were measured in duplicate. To normalize plate-by-plate variations, every ELISA plate included a pooled serum samples in duplicate. The CVs of intra-assay and inter-assay for each autoantibody were below 6% and 10%, respectively.

**References**

Han, C., Jo, S. A., Kim, N. H., Jo, I., & Park, M. H. Study design and methods of the Ansan Geriatric Study (AGE study). *BMC Neurol*. **9**, 10; 10.1186/1471-2377-9-10 (2009).

Shim, S., M., Song, J., Kim, J. H., & Jeon, J. P. Conversion pattern and predictive factor of mild cognitive impairment in elderly Koreans. *Arch Gerontol Geriatr.* **64**, 146-150 (2016).


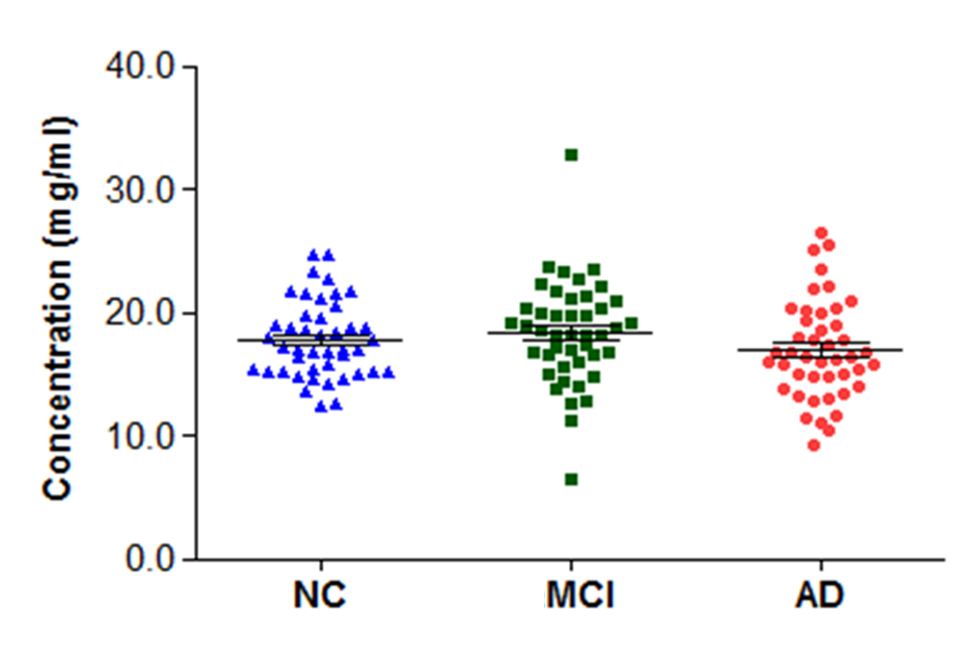


**Supplemental Figure 1. Levels of serum total IgG in the elderly Koreans among AD, MCI and NC groups**. Total IgG levels were not significantly different among NC, MCI, and AD (p=0.227, one-way ANOVA test). These experiments were repeated twice. Each dot represents one subject. Bars show mean ± standard error of the mean (SEM).


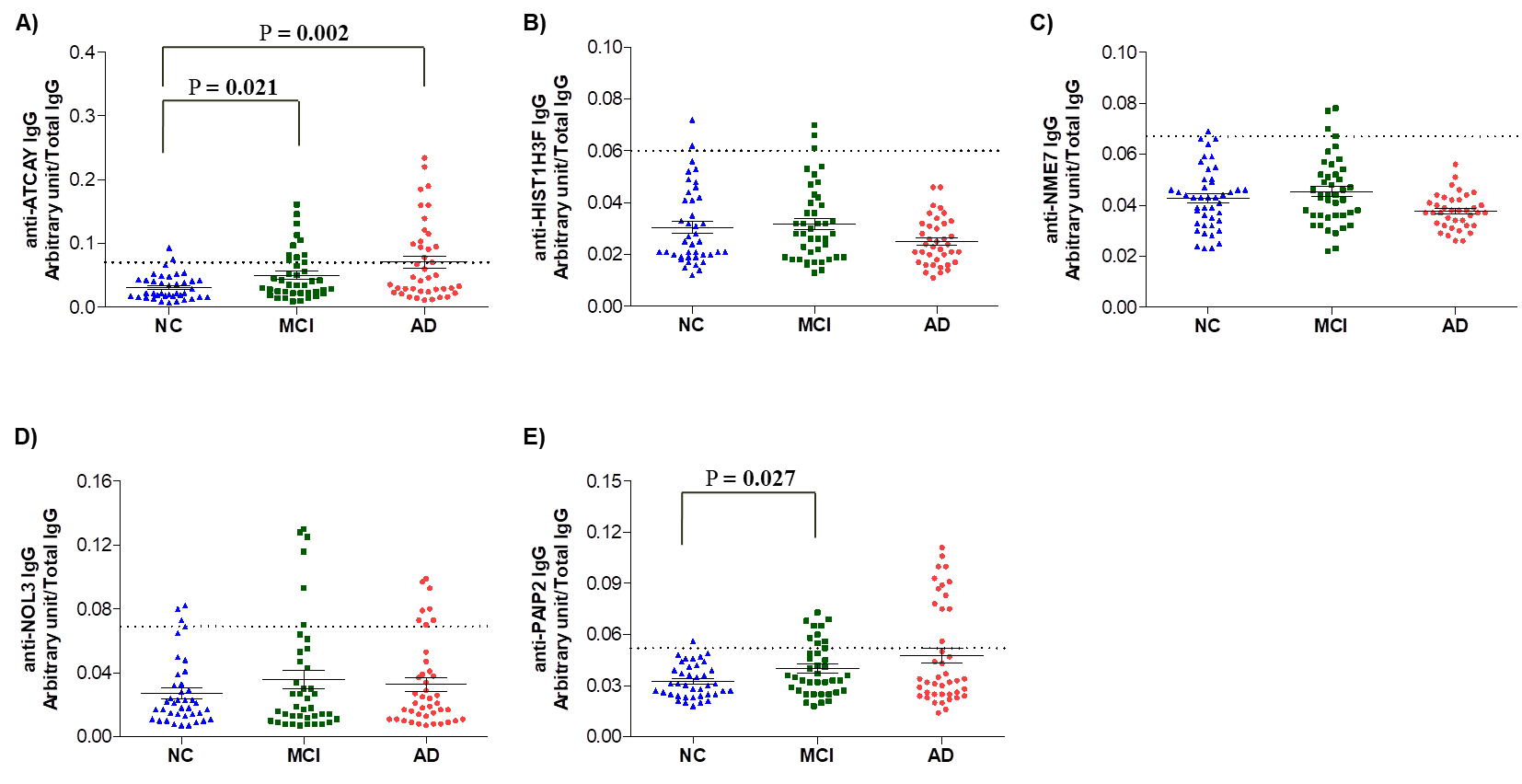


Supplemental Figure 2. Levels of total IgG-normalized autoantibodies in the AD, MCI and cognitively normal control subjects. Levels(AU) of anti-ATCAY IgG (A), anti-HIST1H3F IgG (B), anti-NME7 IgG (C), anti-NOL3 IgG (D), and anti-PAIP2 IgG (E) were normalized by the concentration of total IgG for each participant. Levels of total IgG-normalized anti-ATCAY IgG were higher in the MCI group (p=0.021) and AD (p=0.002) than in the NC group (A). Levels of total IgG-normalized anti-PAIP2 IgG were higher in the MCI group (p=0.027) than in the NC group (E). Statistical significance was calculated by Mann-Whitney test after removing outliers that were more than 1.5 × interquartile range (IQR). Bars show mean ± standard error of the mean (SEM). Each dot represents one participant. Horizontal dotted lines depict the cutoff for the positivity of each autoantibody, which is set at mean plus 2 standard deviation (SD) of autoantibody measurements in the NC group.


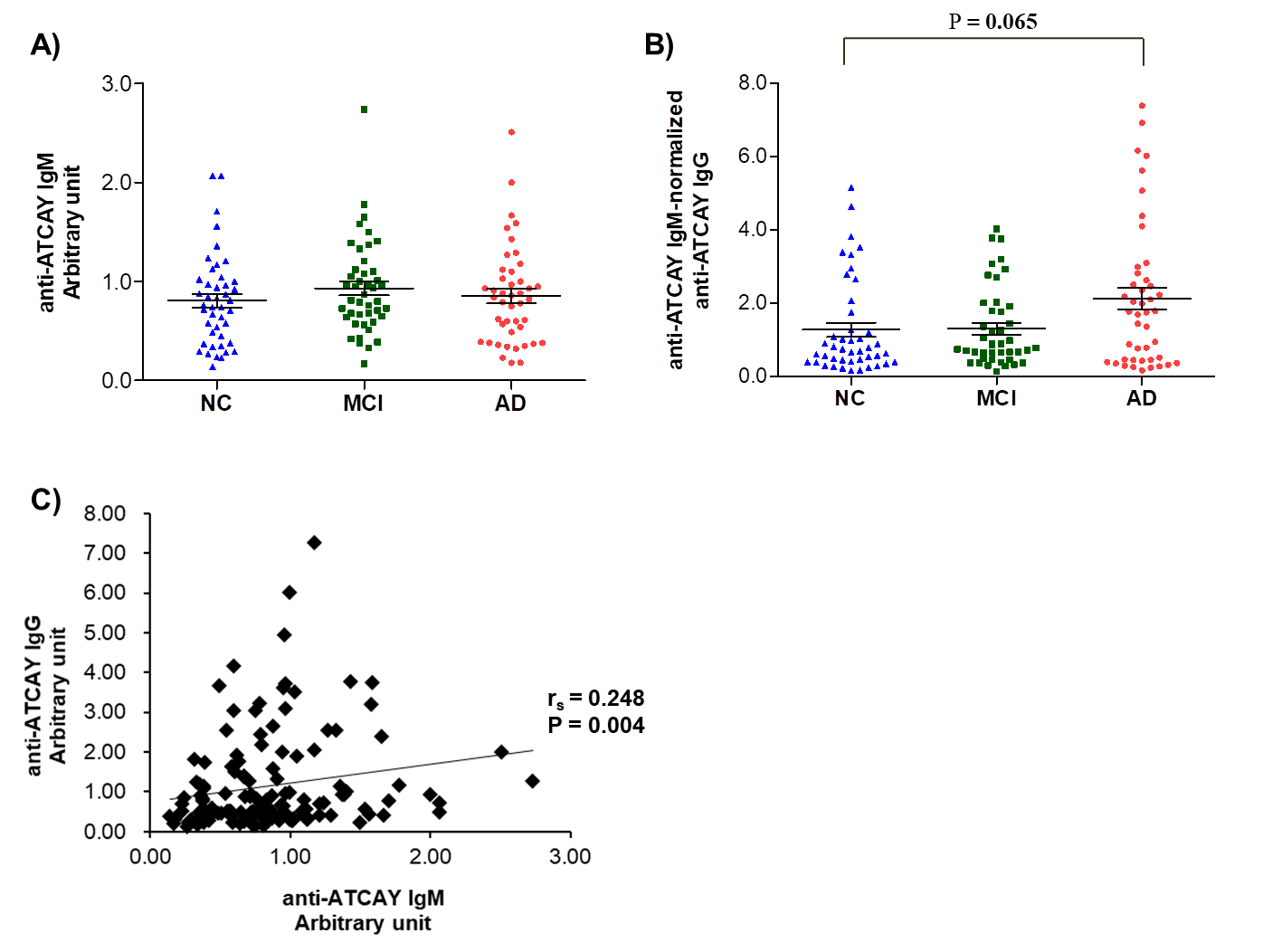


**Supplemental Figure 3. Comparison of anti-ATCAY IgM autoantibody among AD, MCI and NC group and correlation of anti-ATCAY IgM with anti-ATCAY IgG.** Levels of anti-ATCAY IgM autoantibody were not significantly different among three groups (p=0.346, Kruskal-Wallis test) (A). Anti-ATCAY IgM-normalized anti-ATCAY IgG level was marginally significantly higher in the AD than the NC (p=0.065, Mann-Whitney test) (B). Levels of anti-ATCAY IgG autoantibody were positively correlated with anti-ATCAY IgM autoantibody (r_s_=0.248, p=0.004) (C). Each dot represents one subject. Bars show mean ± standard error of the mean (SEM). The correlation was evaluated by Spearman correlation test.

**Supplemental Table 1. Ubiquitous autoantigen candidates in the Korean elderly**

| **Database ID** | **Gene symbol** | **Description** | **Occurrence** |
| --- | --- | --- | --- |
|  |  |  | **NC:AD** |
| NM_001009615 | SPANXN2 | SPANX family, member N2 | 5:5 |
| NM_052957.2 | ACRC | acidic repeat containing | 5:5 |
| BC062740.1 | TPM3 | tropomyosin 3 | 5:5 |
| NM_198505 | ATP13A5 | ATPase type 13A5 | 5:5 |
| BC031259.1 | LOC649897 | ELK2A, member of ETS oncogene family, pseudogene | 5:5 |
| NM_145290 | GPR125 | G protein-coupled receptor 125 | 4:4 |
| ZNF768 | ZNF768 | zinc finger protein 768 | 4:4 |
| NM_033064.3 | ATCAY | ataxia, cerebellar, Cayman type | 4:4 |
| Nol3 | Nol3 | nucleolar protein 3 (apoptosis repressor with CARD domain) | 4:4 |
| NM_001828.5 | CLC | Charcot-Leyden crystal galectin | 3:4 |
| TLK1 | TLK1 | tousled-like kinase 1 | 3:4 |
| BC000267.1 | GPBP1 | GC-rich promoter binding protein 1 | 4:3 |
| NM_019088.2 | PAF1 | Paf1, RNA polymerase II associated factor, homolog (S. cerevisiae) | 4:3 |

**Supplemental Table 2. Abundant autoantigens in AD patients**

| **No.** | **Database ID** | **Gene symbol** | **Description** | **Occurrence^a^ NC:AD** |
| --- | --- | --- | --- | --- |
| 1 | BC039337.1 | PAIP2 | poly(A) binding protein interacting protein 2 | 1:4 |
| 2 | HIST1H3F | HIST1H3F | histone cluster 1, H3f | 1:4 |
| 3 | BX640997 | C4orf40 | proline rich 27 | 0:3 |
| 4 | NM_133640.3 | SURF5 | mediator complex subunit 22 | 0:3 |
| 5 | NM_001080830 | PRAMEF12 | PRAME family member 12 | 3:5 |
| 6 | NM_016307.3 | PRRX2 | paired related homeobox 2 | 2:4 |
| 7 | NM_003946.3 | NOL3 | nucleolar protein 3 (apoptosis repressor with CARD domain) | 2:4 |
| 8 | NM_001008708.1 | CHAC2 | ChaC, cation transport regulator homolog 2 (E. coli) | 2:4 |
| 9 | NM_004677 | XKRY | XK, Kell blood group complex subunit-related, Y-linked | 2:4 |
| 10 | BC063137.1 | PDK2 | pyruvate dehydrogenase kinase, isozyme 2 | 2:4 |
| 11 | NM_001005922 | KRTAP5-1 | keratin associated protein 5-1 | 1:3 |
| 12 | NM_001002233.1 | RAB11FIP1 | RAB11 family interacting protein 1 (class I) | 1:3 |
| 13 | NM_198507.1 | TMEM157 | family with sequence similarity 174, member A | 1:3 |
| 14 | NM_004791.2 | ITGBL1 | integrin, beta-like 1 (with EGF-like repeat domains) | 1:3 |
| 15 | BC024289.1 | IFI6 | interferon, alpha-inducible protein 6 | 1:3 |
| 16 | BC032657 | TLK1 | tousled-like kinase 1 | 1:3 |
| 17 | UBXN2B | UBXN2B | UBX domain protein 2B | 0:2 |
| 18 | NM_021959.2 | PPP1R11 | protein phosphatase 1, regulatory (inhibitor) subunit 11 | 0:2 |
| 19 | NM_001030312.2 | CERKL | ceramide kinase-like | 0:2 |
| 20 | NM_001009941.1 | ANKRD16 | ankyrin repeat domain 16 | 0:2 |
| 21 | DKK2 | DKK2 | dickkopf WNT signaling pathway inhibitor 2 | 0:2 |
| 22 | NM_182498.2 | ZNF428 | zinc finger protein 428 | 0:2 |
| 23 | NM_003685.2 | KHSRP | KH-type splicing regulatory protein | 0:2 |
| 24 | BC050132 | TXNDC2 | thioredoxin domain containing 2 (spermatozoa) | 0:2 |
| 25 | NM_014301.2 | ISCU | iron-sulfur cluster assembly enzyme | 0:2 |
| 26 | NM_017871.4 | CPSF3L | cleavage and polyadenylation specific factor 3-like | 0:2 |
| 27 | NM_007045.2 | FGFR1OP | FGFR1 oncogene partner | 0:2 |
| 28 | NM_001726.3 | BRDT | bromodomain, testis-specific | 0:2 |
| 29 | ZNF540 | ZNF540 | zinc finger protein 540 | 0:2 |
| 30 | NM_032593.2 | HINT2 | histidine triad nucleotide binding protein 2 | 0:2 |
| 31 | NM_002242.2 | KCNJ13 | potassium inwardly-rectifying channel, subfamily J, member 13 | 0:2 |
| 32 | NM_022456.3 | RAB3IP | RAB3A interacting protein | 0:2 |
| 33 | ST8SIA1 | ST8SIA1 | ST8 alpha-N-acetyl-neuraminide alpha-2,8-sialyltransferase 1 | 0:2 |
| 34 | NM_001080500.2 | VWC2L | von Willebrand factor C domain containing protein 2-like | 0:2 |
| 35 | BC056249.1 | PSMA4 | proteasome (prosome, macropain) subunit, alpha type, 4 | 0:2 |
| 36 | NM_003681.3 | PDXK | pyridoxal (pyridoxine, vitamin B6) kinase | 0:2 |
| 37 | BC005934.1 | SIKE | suppressor of IKBKE 1 | 0:2 |
| 38 | NM_148975.1 | MS4A4A | membrane-spanning 4-domains, subfamily A, member 4A | 0:2 |
| 39 | NM_024506.3 | GLB1L | galactosidase, beta 1-like | 0:2 |
| 40 | NM_152237.1 | GAS2L1 | growth arrest-specific 2 like 1 | 0:2 |
| 41 | NM_031964 | KRTAP17-1 | keratin associated protein 17-1 | 0:2 |
| 42 | NM_003099.3 | SNX1 | sorting nexin 1 | 0:2 |
| 43 | NM_032905.3 | RBM17 | RNA binding motif protein 17 | 0:2 |
| 44 | NM_014622.4 | LOH11CR2A | von Willebrand factor A domain containing 5A | 0:2 |
| 45 | NM_139164.1 | STARD4 | StAR-related lipid transfer (START) domain containing 4 | 0:2 |
| 46 | NM_020696.1 | KIAA1143 | KIAA1143 | 0:2 |
| 47 | NM_175634.1 | RUNX1T1 | runt-related transcription factor 1; translocated to, 1 (cyclin D-related) | 0:2 |

^a^ The autoantigen occurrence is shown in the case that a particular autoantigen exhibited occurrence difference (Delta) between AD and NC is more than 2.

**Supplemental Table 3. Enrichment analysis of AD-abundant autoantigens^a^ using DAVID**

| **Cluster** | **Enrichment**  **score^b^** | **Category** | **Term** | **Count** | ***p-value^c^*** | **Benjamini** |
| --- | --- | --- | --- | --- | --- | --- |
| 1 | 4.12 | GOTERM_CC_FAT | GO:0043228~non-membrane-bounded organelle | 53 | 2.71E-05 | 7.32E-03 |
|  |  | GOTERM_CC_FAT | GO:0043232~intracellular non-membrane-bounded organelle | 53 | 2.71E-05 | 7.32E-03 |
|  |  | GOTERM_CC_FAT | GO:0005856~cytoskeleton | 31 | 6.12E-04 | 7.96E-02 |
| 2 | 2.28 | GOTERM_BP_FAT | GO:0008104~protein localization | 23 | 4.52E-04 | 4.69E-01 |
|  |  | GOTERM_BP_FAT | GO:0015031~protein transport | 20 | 1.14E-03 | 5.49E-01 |
|  |  | GOTERM_BP_FAT | GO:0045184~establishment of protein localization | 20 | 1.27E-03 | 4.46E-01 |
|  |  | GOTERM_BP_FAT | GO:0034613~cellular protein localization | 13 | 2.74E-03 | 5.36E-01 |
|  |  | GOTERM_BP_FAT | GO:0070727~cellular macromolecule localization | 13 | 2.91E-03 | 4.92E-01 |
|  |  | SP_PIR_KEYWORDS | protein transport | 14 | 3.58E-03 | 1.50E-01 |
|  |  | GOTERM_BP_FAT | GO:0046907~intracellular transport | 16 | 8.43E-03 | 5.46E-01 |
|  |  | GOTERM_BP_FAT | GO:0006886~intracellular protein transport | 11 | 1.10E-02 | 5.56E-01 |
| 3 | 1.93 | GOTERM_MF_FAT | GO:0030674~protein binding, bridging | 8 | 1.10E-04 | 4.06E-02 |
|  |  | BIOCARTA | h_metPathway:Signaling of Hepatocyte Growth Factor Receptor | 5 | 1.68E-03 | 1.35E-01 |
|  |  | GOTERM_MF_FAT | GO:0005070~SH3/SH2 adaptor activity | 5 | 2.48E-03 | 3.74E-01 |
|  |  | GOTERM_MF_FAT | GO:0060090~molecular adaptor activity | 5 | 7.64E-03 | 6.19E-01 |
|  |  | UP_SEQ_FEATURE | domain:SH2 | 5 | 2.39E-02 | 9.22E-01 |
|  |  | INTERPRO | IPR000980:SH2 motif | 5 | 4.15E-02 | 9.97E-01 |
|  |  | SMART | SM00252:SH2 | 5 | 4.56E-02 | 8.91E-01 |
| 4 | 1.75 | SP_PIR_KEYWORDS | sh3 domain | 8 | 9.96E-03 | 3.23E-01 |
|  |  | INTERPRO | IPR001452:Src homology-3 domain | 8 | 1.28E-02 | 9.95E-01 |
|  |  | SMART | SM00326:SH3 | 8 | 1.47E-02 | 7.55E-01 |

^a^ The autoantigens used in this analysis includes all autoantigens with higher occurrence in AD comparing to NC.

^b^ Enrichment score > 1.5

^c^ p-value < 0.05

**Supplemental Table 4. Enriched KEGG pathway associated with AD-abundant autoantigens^a^**

| **Term** | **Count** | ***p-value*^b^** | **Benjamini** |
| --- | --- | --- | --- |
| ErbB signaling pathway | 7 | 4.18E-04 | 4.42E-02 |
| Neurotrophin signaling pathway | 7 | 2.67E-03 | 1.35E-01 |
| Focal adhesion | 8 | 7.37E-03 | 2.34E-01 |
| Pathways in cancer | 10 | 1.11E-02 | 2.60E-01 |
| Insulin signaling pathway | 6 | 1.82E-02 | 3.28E-01 |

^a^ The autoantigens used in this analysis includes all autoantigens with higher occurrence in AD comparing to NC.

^b^ p-value < 0.05

**Supplemental Table 5**. **Correlation of measurements of target autoantibodies between protein microarray and ELISA experiments**

| **Autoantibodies** | **r_s_** | ***p-value*** |
| --- | --- | --- |
| anti-ATCAY IgG | **0.733** | **0.016** |
| anti-CHAC2 IgG | 0.527 | 0.117 |
| anti-CLC IgG | 0.097 | 0.789 |
| anti-GPBP1 IgG | 0.612 | 0.060 |
| anti-HIST1H3F IgG | **0.772** | **0.009** |
| anti-NME7 IgG | **0.717** | **0.020** |
| anti-NOL3 IgG | **0.770** | **0.009** |
| anti-PAIP2 IgG | **0.650** | **0.042** |
| anti-RAB11FIP1 IgG | 0.455 | 0.187 |
| anti-SPANXN2 IgG | 0.479 | 0.162 |
| anti-SURF5 IgG | -0.188 | 0.603 |
| anti-TPM3 IgG | 0.261 | 0.467 |

Spearman correlation analysis was estimated for correlation between chip data and ELISA AU.

**Supplemental Table 6. Frequency of ApoE alleles in the discovery and validation sets**

| ApoE genotypes | Discovery set | |  | Validation set | | |
| --- | --- | --- | --- | --- | --- | --- |
|  | NC (%) | AD (%) |  | NC (%) | MCI (%) | AD (%) |
| e2/e3 | - | - |  | 16.3 | 11.9 | 6.8 |
| e2/e4 | 20.0 | - |  | 2.3 | 2.4 | - |
| e3/e3 | 60.0 | - |  | 72.1 | 57.1 | 52.3 |
| e3/e4 | 20.0 | 80.0 |  | 9.3 | 26.2 | 38.6 |
| e4/e4 | - | 20.0 |  | - | 2.4 | 2.3 |

**Supplemental Table 7. ApoE4-dependent difference in the autoantibody levels in the validation set**

| Autoantibodies | Non-ApoE4 carriers  (n=96) | ApoE4 carriers  (n=36) | *p-value^a^* |
| --- | --- | --- | --- |
| anti-ATCAY IgG | 1.13 ± 1.25 | 1.26 ± 1.23 | 0.351 |
| anti-HIST1H3F IgG | 0.63 ± 0.57 | 0.66 ± 0.59 | 0.925 |
| anti-NME7 IgG | 0.76 ± 0.29 | 0.83 ± 0.50 | 0.988 |
| anti-NOL3 IgG | 0.81 ± 0.89 | 0.56 ± 0.62 | 0.121 |
| **anti-PAIP2 IgG** | **0.81 ± 0.66** | **1.11 ± 1.23** | **0.049** |

Data are shown as mean ± standard deviation (SD).

^a^ Significant differences were evaluated by Mann-Whitney U test.

**Supplemental Table 8. Gender-dependent difference in the autoantibody levels the in the validation set**

| Autoantibodies | All (n=132) | | |  | NC (n=44) | | |  | MCI (n=44) | | |  | AD (n=44) | | |
| --- | --- | --- | --- | --- | --- | --- | --- | --- | --- | --- | --- | --- | --- | --- | --- |
|  | Male  (n=24) | Female  (n=108) | *p-value^a^* |  | Male  (n=8) | Female (n=36) | *p-value^a^* |  | Male  (n=8) | Female (n=36) | *p-value^a^* |  | Male  (n=8) | Female (n=36) | *p-value^a^* |
| anti-ATCAY IgG | 0.90 ± 0.85 | 1.22 ± 1.31 | 0.585 |  | 0.61 ± 0.62 | 0.93 ± 1.09 | 0.338 |  | 0.80 ± 0.40 | 1.16 ± 1.00 | 0.659 |  | 1.28 ± 1.25 | 1.58 ± 1.68 | 0.843 |
| anti-HIST1H3F IgG | 0.49 ± 0.26 | 0.67 ± 0.62 | 0.110 |  | 0.48 ± 0.28 | 0.73 ± 0.76 | 0.212 |  | 0.64 ± 0.26 | 0.63 ± 0.44 | 0.474 |  | **0.34 ± 0.16** | **0.66 ± 0.63** | **0.037** |
| anti-NME7 IgG | 0.73 ± 0.24 | 0.80 ± 0.39 | 0.439 |  | 0.71 ± 0.16 | 0.76 ± 0.27 | 0.692 |  | 0.89 ± 0.29 | 0.85 ± 0.35 | 0.553 |  | 0.59 ± 0.19 | 0.78 ± 0.49 | 0.075 |
| anti-NOL3 IgG | 0.51 ± 0.54 | 0.80 ± 0.88 | 0.130 |  | 0.67 ± 0.81 | 0.71 ± 0.72 | 0.692 |  | 0.54 ± 0.37 | 0.97 ± 1.14 | 0.915 |  | **0.33 ± 0.34** | **0.71 ± 0.70** | **0.041** |
| anti-PAIP2 IgG | 1.12 ± 1.48 | 0.84 ± 0.64 | 0.684 |  | 0.72 ± 0.15 | 0.72 ± 0.55 | 0.077 |  | 1.40 ± 1.97 | 0.90 ± 0.63 | 0.843 |  | 1.26 ± 1.74 | 0.90 ± 0.74 | 0.503 |

Data are shown as mean ± standard deviation (SD).

^a^ Significant differences were evaluated by Mann-Whitney U test.

**Supplemental Table 9. Levels of anti-Tau IgG in AD, MCI and cognitively normal control (NC).**

| Autoantibodies | NC  (n=24) | MCI  (n=24) | AD  (n=24) | *p-value^a^* |
| --- | --- | --- | --- | --- |
| Anti-tTau IgG | 0.87 ± 0.73 | 1.01 ± 0.83 | 0.88 ± 0.72 | 0.109 |
| Anti-pTau IgG | 0.80 ± 0.40 | 0.86 ± 0.43 | 0.80 ± 0.40 | 0.894 |

Data are shown as mean ± standard deviation (SD).

^a^ Significant differences were evaluated by Kruskal-Wallis test.

**Supplemental Table 10. Correlation of levels of autoantibodies and the concentration of total IgG**

| **Autoantibodies** | **r_s_** | ***p-value*** |
| --- | --- | --- |
| anti-ATCAY IgG | 0.194 | 0.026 |
| anti-HIST1H3F IgG | 0.349 | <0.001 |
| anti-NME7 IgG | 0.473 | <0.001 |
| anti-NOL3 IgG | 0.192 | 0.027 |
| anti-PAIP2 IgG | 0.265 | 0.002 |

Spearman correlation analysis was estimated for correlation between autoantibody AU and concentration of total IgG.

**Supplemental Table 11. Information of recombinant proteins used in the validation experiments**

| **Recombinant protein** | **Cat. No.** | **Manufacturer** |
| --- | --- | --- |
| ATCAY | TP304515 | OriGene, Rockwille, MD, USA |
| CHAC2 | TP305083 | OriGene, Rockwille, MD, USA |
| CLC | TP319689 | OriGene, Rockwille, MD, USA |
| GPBP1 | H00065056 | Abnova, Taipei, Taiwan |
| HIST1H3F | H00008996 | Abnova, Taipei, Taiwan |
| NME7 | H00029922 | Abnova, Taipei, Taiwan |
| NOL3 | H00008968 | Abnova, Taipei, Taiwan |
| PAIP2 | NBP1-30279 | NOVUS Biologicals, Littleton, CO, USA |
| RAB11FIP1 | H00080223 | Abnova, Taipei, Taiwan |
| SPANXN2 | H00494119 | Abnova, Taipei, Taiwan |
| SURF5 | TP308015 | OriGene, Rockwille, MD, USA |
| TPM3 | TP303276 | OriGene, Rockwille, MD, USA |
